# Therapeutic Angiogenesis by a “Dynamic Duo”: Simultaneous Expression of HGF and VEGF165 by Novel Bicistronic Plasmid Restores Blood Flow in Ischemic Skeletal Muscle

**DOI:** 10.3390/pharmaceutics12121231

**Published:** 2020-12-18

**Authors:** Ekaterina Slobodkina, Maria Boldyreva, Maxim Karagyaur, Roman Eremichev, Natalia Alexandrushkina, Vadim Balabanyan, Zhanna Akopyan, Yelena Parfyonova, Vsevolod Tkachuk, Pavel Makarevich

**Affiliations:** 1 Faculty of Medicine, Lomonosov Moscow State University, 117192 Moscow, Russia; darth_max@mail.ru (M.K.); n.alexandrushkina@gmail.com (N.A.); bal.pharm@mail.ru (V.B.); zhanna.fbm@gmail.com (Z.A.); yeparfyon@mail.ru (Y.P.); tkachuk@fbm.msu.ru (V.T.); pmakarevich@mc.msu.ru (P.M.); 2 Institute for Regenerative Medicine, Medical Research and Education Centre, Lomonosov Moscow State University, 119192 Moscow, Russia; romaneremichev@gmail.com; 3 National Medical Research Center of Cardiology Russian Ministry of Health, 121552 Moscow, Russia; mboldyreva@inbox.ru; 4 Faculty of Biology and Biotechnology, National Research University Higher School of Economics (HSE), 109028 Moscow, Russia

**Keywords:** gene therapy, plasmids, bicistronic vector, angiogenic growth factors, limb ischemia, therapeutic angiogenesis, VEGF, HGF

## Abstract

Therapeutic angiogenesis is a promising strategy for relief of ischemic conditions, and gene delivery was used to stimulate blood vessels’ formation and growth. We have previously shown that intramuscular injection of a mixture containing plasmids encoding vascular endothelial growth factor (VEGF)165 and hepatocyte growth factor (HGF) leads to restoration of blood flow in mouse ischemic limb, and efficacy of combined delivery was superior to each plasmid administered alone. In this work, we evaluated different approaches for co-expression of HGF and VEGF165 genes in a panel of candidate plasmid DNAs (pDNAs) with internal ribosome entry sites (IRESs), a bidirectional promoter or two independent promoters for each gene of interest. Studies in HEK293T culture showed that all plasmids provided synthesis of HGF and VEGF165 proteins and stimulated capillary formation by human umbilical vein endothelial cells (HUVEC), indicating the biological potency of expressed factors. Tests in skeletal muscle explants showed a dramatic difference and most plasmids failed to express HGF and VEGF165 in a significant quantity. However, a bicistronic plasmid with two independent promoters (cytomegalovirus (CMV) for HGF and chicken b-actin (CAG) for VEGF165) provided expression of both grow factors in skeletal muscle at an equimolar ratio. Efficacy tests of bicistronic plasmid were performed in a mouse model of hind limb ischemia. Intramuscular administration of plasmid induced significant restoration of perfusion compared to an empty vector and saline. These findings were supported by increased CD31+ capillary density in animals that received pHGF/VEGF. Overall, our study reports a first-in-class candidate gene therapy drug to deliver two pivotal angiogenic growth factors (HGF and VEGF165) with properties that provide basis for future development of treatment for an unmet medical need—peripheral artery disease and associated limb ischemia.

## 1. Introduction

Therapeutic angiogenesis is an approach to restore tissue supply and relieve ischemia by gene delivery in vivo to express angiogenic growth factors (AGF) stimulating blood vessel growth. This strategy has been proposed for treatment of conditions caused by impaired circulation—e.g., chronic lower limb ischemia, diabetic neuropathy, coronary heart disease and myocardial infarction. In recent decades, development of therapeutic angiogenesis using plasmid DNA (pDNA) encoding AGF genes resulted in marketing of gene therapy drugs (“Neovasculgen”, Russia; “Collategene”, Japan) and remains an important field in translational research. However, in clinical trials, the majority of non-viral gene therapy drugs encoding vascular endothelial growth factor (VEGF165), hepatocyte growth factor (HGF) or basic fibroblast growth factor (bFGF) demonstrated plausible safety profiles with limited efficacy in patients with cardiovascular disease [1,2,3].

Accumulated data also proposed that delivery of a single AGF is not sufficient to render a long-term positive effect in patients with advanced stages of ischemic disease. Angiogenesis and accompanying nerve growth are multi-staged processes regulated by various cytokines, proteases and growth factors—therefore, a sole factor delivered as a recombinant protein or genetic construct may fail to induce a durable impact. A potential solution is the selection and use of physiologically relevant combinations of factors that may render potential additive effects.

Experimental studies in animal models of ischemic disease, including our works, show that delivery of two angiogenic or tissue-protective molecules may substantially enhance efficacy. Combined gene delivery of VEGF165 with angiopoietin-1 [4], bFGF [5], urokinase plasminogen activator (uPA) [6], stromal-derived factor-1α (SDF) [7] or HGF [8,9] has shown increased angiogenic response compared to these factors delivered alone.

Our group has demonstrated the efficacy of intramuscular injection of a mixture of independent plasmids encoding VEGF165 and HGF for stimulation of angiogenesis. Administration of a mixture of pDNAs with VEGF165 and HGF genes to mice with hind limb ischemia resulted in enhanced angio- and arteriogenesis and decreased necrosis in skeletal muscle compared to each factor alone [8]. Similar effect of plasmids mixture was also shown in a rat myocardial ischemia model. Administration of a combination of plasmids with VEGF165 and HGF did not exceed single plasmids in stimulating arteriogenesis or reducing myocardial damage; however, it more efficiently stimulated angiogenesis in myocardium compared to using a plasmid with a single AGF [9]. These results supported the efficacy of combined use of these AGFs and potential development of a gene therapy drug. Unfortunately, the use of a pDNAs mixture does not allow to obtain reproducible kinetics, so there is a need to develop a single-molecule combined gene therapy drug for simultaneous expression in impaired tissue.

Disadvantages of injecting a mixture of vectors include unpredictable probability of co-transfection by several plasmids. It is marginally impossible to predict the rate of transfection by each vector to describe pharmacokinetics and ensure a stable profile of local growth factor concentrations after injection of a mixture of several pDNAs. Furthermore, complex stoichiometry and differences in expression may result in loss of additive effects expected in combined approaches [10].

**Figure 1:**
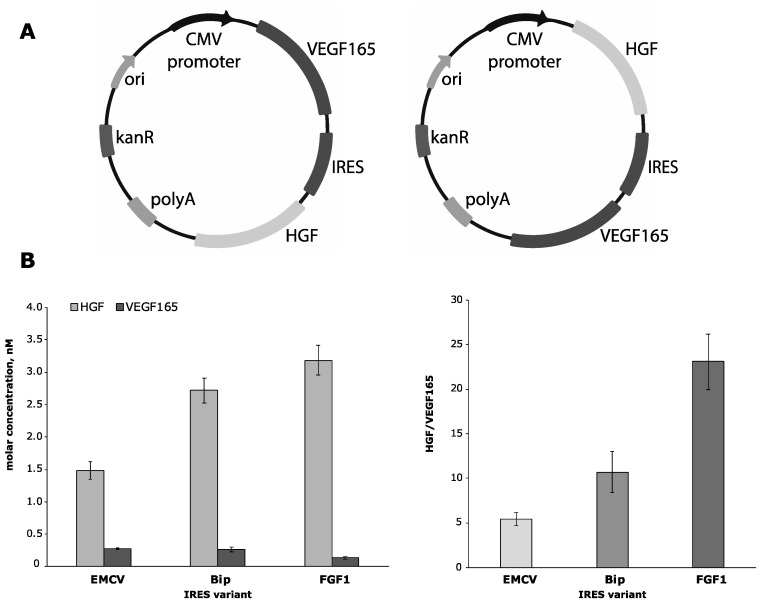
Designs of internal ribosome entry site (IRES)-based cytomegalovirus (CMV)-driven plasmids and results of expression activity analysis in HEK293T culture. (**A**) IRES-based plasmid vectors to express human hepatocyte growth factor (HGF) and vascular endothelial growth factor (VEGF)165 with alternative designs: (**left**) VEGF165 gene upstream to EMCV_IRES or (**right**) HGF gene upstream to EMCV_IRES. (**B**) Molar concentrations of HGF and VEGF165 (**left**) and their ratios (**right**) after ELISA of culture medium from transfected HEK293T cells. Data presented as mean ± SEM. Results are only from samples with both detectable angiogenic growth factors (AGFs) provided.

All this complicates pharmaceutical development of drugs composed by a combination of two or more pDNA constructs. A promising way to overcome these limitations is development of genetic constructs that allow simultaneous delivery of two or more genes via a polycistronic coding vector. General strategies to achieve this goal include potential designs of plasmid DNA with the following elements to provide simultaneous expression of several AGFs (see Figure 1).

(1) Vectors with an “internal” promoter where each encoded gene has its independent enhancer, promoter and polyA signal [11]. This design allows selection of appropriate promoters depending on the type of transfected tissue and impairing condition (hypoxia, inflammation and concomitant chemotherapy). Nevertheless, uncoupled transcription of genes can occur in such vectors due to promoter suppression by cross-influence of translation products after gene delivery [12,13].

(2) Bidirectional promoters that can be cloned from human and other species’ genomes. These provide transcription of two genes in opposite directions of a DNA strand. A bidirectional promoter must be “strong” and provide high transcriptional activity in different types of cells and must be resistant to silencing by epigenetic factors [14,15]. Typical bidirectional promoters are phosphoglycerate kinase (PGK1) and chicken β-actin (CAG) gene promoters, as well as artificial ones generated by fusion of the translational elongation factor 1 human alpha (EEF1A1) promoter with minimal sequences of cytomegalovirus (minCMV), or Pgk1 with cytomegalovirus promoter [16].

(3) Genes can be separated by an internal ribosomal entry site (IRES)—a sequence that ensures binding of ribosome subunits to mRNA and start of protein synthesis without involving the 5′-end of the mRNA. In such constructs, both coding sequences are transcribed under one promoter as a single transcript with genes of interest separated by an IRES. Translation of the gene located upstream of the IRES occurs by a classical cap-dependent mechanism while translation of the second gene is initiated by the IRES in a cap-independent (internal) manner. The most commonly used IRES is cloned from encephalomyocarditis virus (EMCV). The main disadvantage of this design is the reduced expression of the downstream gene which is generally several-fold lower than that of upstream gene [17,18,19,20]. The efficacy of IRES-driven translation will depend on the IRES used as well as the type of vector and transfected cell type [19,21,22]. In addition, the IRES accounts several hundred b.p. so its use can limit the size of cloned genes due to vector capacity.

Current study reports on experimental development of a candidate pDNA-based drug to express HGF and VEGF165 in mammalian tissue and evaluation of its therapeutic efficacy in a mouse model of limb ischemia. We believe that the results of this work are of interest for development of novel gene therapy drugs for more efficient treatment of limb ischemia and other cardiovascular diseases associated with impaired blood supply.

## 2. Materials and Methods

### 2.1. Plasmid Vector Design and Purification

Codon-optimized complementary DNA (cDNA) of human HGF and VEGF165 was cloned to a nonoptimized first generation DNA vaccine vector (pVAX)-based plasmid.

Three different types of bicistronic vectors were created:

1. Plasmids carrying different variants of IRES to ensure simultaneous expression of HGF and VEGF165 genes (general structure schemes: “pCMV_HGF-[IRES]–VEGF165”). We used IRES sequences cloned from EMCV, binding immunoglobulin protein (Bip), FGF1 and eukaryotic translation initiation factor 4 G (elF4G) genes (see Supplementary S1).
2. A vector with the bidirectional cytomegalovirus (CMV) promoter common for the HGF and VEGF165 genes (bi-HGF/VEGF).
3. A vector with two independent promotors: CMV for HGF and CAG for VEGF165 genes (pHGF/VEGF).

Plasmid DNA with green fluorescent protein (GFP) gene driven by CMV promoter was used to assure transfection in cell cultures in separate samples. All plasmids were amplified in *Escherichia coli* (DH-5α strain) and purified using the EndoFree Plasmid Giga Kit following the manufacturer’s instructions (Qiagen).

### 2.2. Cell Culture and Ca^2+^/Phosphate Transfection

For in vitro plasmid testing, we used HEK293T cells and the Ca^2+^/phosphate transfection method, which was described earlier [8,9]. HGF and VEGF165 concentrations were evaluated by Quantikine Elisa kits for VEGF165 (DVE00) or HGF (DHG00), both from R&D Systems.

#### 2.2.1. Tube Formation Assay

Matrigel (356231, Corning, NY, USA) was thawed according to the manufacturer’s instructions. Wells of a 48-well plate were filled with 200 μL of Matrigel and incubated for 1 h at +4 °C; thereafter, the excess of Matrigel was removed from the wells. Then, the plate was incubated for 15 min in an incubator at 37 °C for polymerization of the Matrigel. Human umbilical vein endothelial cells (HUVECs) were taken at passage 3 and were cultured in EGM-2 (Lonza, Basel, Switzerland) until 80% confluence. Then, the cells were detached from plastic with 0.05% trypsin-EDTA solution (Gibco, Waltham, MA, USA), centrifuged at 300× *g* for 5 min and resuspended in 200 μL of EBM-2 (Lonza, Basel, Switzerland). After cells’ quantification, the suspension was additionally diluted up to a concentration of 5 million cells/mL, and 50 × 10^3^ cells (10 μL of the suspension) were added to tested and control medium prepared for assay (200 μL for each plate well). Conditioned medium from mock-transfected HEK293T (with 0.5% FBS), Dulbecco’s Modified Eagle Medium (DMEM) (Life Technologies, Carlsbad, CA, USA) without fetal bovine serum (FBS) and DMEM with 0.5% FBS (Hyclone, Logan, UT, USA) were used as negative controls. EGM-2 was used as a positive control. Obtained suspensions were added to the wells with Matrigel thereafter.

An IncuCyte^®^ Live-Cell analysis system (Essen BioScience, Ann Arbor, MI, USA) was used for experiments with IRES-based plasmids. Microphotographs were taken in phase contrast mode with 100× magnification every 2 h. Tube length and branching points numbers were analyzed using Angiogenesis Analyzer of ImageJ software (NIH, Bethesda, MD, USA) in 5 randomly selected fields of view (FOV) taken from each plate well, and mean values for the control substance or tested medium sample were obtained.

#### 2.2.2. Tube Assay with Assessment of Cells Mortality and Survival

Matrigel and HUVEC preparations were performed as described earlier. Suspensions of cells in the control or tested medium were added to the Matrigel-coated 48-well plate. The plate was transferred to the incubator of the BioSpa Live Cell Analysis System (BioTek, Winooski, VT, USA). Every 4 h, using 4× objective and the same protocol, the system performed panoramic imaging in bright field (12 fields of view for each well with their subsequent stitching). Through 24 h after the beginning of the experiment, 7 μL of a mixture consisting of 5 μL of 0.5 mM Hoechst 33342 (Thermo Fisher Scientific, Waltham, MA, USA) and 2 μL of 1 mM ethidium homodimer (Sigma-Aldrich, St. Louis, MO, USA) were added directly to the wells for visualization of living and dead cells (Hoechst 33342 marked nuclei of all cells; ethidium homodimer—dead cells only). The plate was incubated for 5 min and then imaging was performed in the same fields of view as earlier with addition of 4′,6-diamidino-2-phenylindole (DAPI) and red fluorescent protein (“RFP”) channels for detection of fluorescent signal from Hoechst 33342 and ethidium homodimer, respectively. After completion of the imaging, which lasted about 1 h, the plate wells were washed once with DMEM (Gibco, USA) with 0.5% FBS (Hyclone, USA) to discard most of the dead cells and imaging was performed again in the same fields of view with the same settings. Images of capillary-like structures obtained in bright field were analyzed in ImageJ using Angiogenesis Analyzer. The calculation of the total area (sum area) of objects labeled with Hoechst 33342 and/or ethidium homodimer was done using the Gen5 software (BioTek, USA). The cell survival index was determined by the following formula: ∑*_area Hoechst_* − ∑*_area ethidium homodimer_*.

#### 2.2.3. Tube Assay with Assessment of Cells Apoptosis

A tube assay for apoptosis assessment was performed in a 96-well plate. Manipulations with Matrigel were performed as described above; 20 × 10^3^ HUVECs and 100 μL of medium per well were used. Incucyte^®^ Caspase-3/7 Green Dye (4440, eBioScience, Ann Arbor, MI, USA) at a final concentration of 5 μM was added to final cell suspensions and was used for apoptosis evaluation. Every 4 h, using 4× objective and the same protocol, the BioSpa Live Cell Analysis System (BioTek, USA) performed panoramic imaging in bright field and “GFP” channel (4 fields of view for each well with their subsequent stitching). The sum area occupied by the cells was calculated from bright field images, and the sum area of activated Incucyte^®^ Caspase-3/7 Green Dye–from GFP channel images. Relative caspase-3 activity was determined by the following formula: caspase-3/7 dye sum area/cells sum area.

#### 2.2.4. Animal Strain and Ethical Approval

Eight-week-old C57BL/6 male mice were used for hind limb ischemia experiments and ex vivo detection of transgene expression. All animals received standard food and water ratios. Surgical manipulations and euthanasia protocols were designed in accordance to Institute and National regulations. All animal experiments were carried out in compliance with internal “Rules for conducting work using experimental animals” and were approved by the Ethical Board of the National Medical Research Center of Cardiology (permit # 16-10-00).

### 2.3. Plasmid Injection and Low-Voltage Electroporation

For intramuscular administration of plasmids, sterile pDNA solutions in 0.9% NaCl were used. Animals received intramuscular injection (in m. tibialis anterior) of 100 µg of the experimental plasmid or a similar volume of physiological saline (negative control).

For explanted muscle tests (see below) of IRES-based plasmids, we used electroporation as described by Schertzer and Lynch [23] to increase the efficiency of transfection with minimal modification; we omitted injection of hyaluronidase, described in the original method. Immediately after the injection of plasmids on the skin adjacent to the m. tibialis anterior, plate electrodes covered by conductive gel were applied. Electroporation was performed by three pulses with a voltage of 80 V/cm of the distance between the electrodes, a frequency of 1 Hz and a duration of 20 ms using a constant current pulse generator of the BTX-Harvard apparatus ECM 830 brand (Harvard apparatus, Holliston, MA, USA). Then, the polarity of the electrodes changed and 3 more pulses with similar reduced characteristics were supplied. The field of electroporation was cleaned with 70% ethanol. Explanted muscle tests of bi-HGF/VEGF and pHGF/VEGF plasmids were performed without electroporation.

### 2.4. Ex Vivo Analysis of HGF and VEGF165 Production by Explanted Muscle

Skeletal muscle samples from animals injected with experimental plasmids were harvested at day 2 after injection and used for generation skeletal muscle explant culture. Skeletal muscle explant culture experiments were performed in accordance with the protocol described by Jang and Kim [24]. Skeletal muscle samples were placed in Matrigel and then cultured in complete DMEM with 4.5 g/L glucose (Life Technologies, USA) and 2% FBS (Gibco, USA) under standard conditions. Culture medium samples were harvested on the 3rd and 6th days in culture. HGF and VEGF165 concentrations were evaluated by ELISA as described above.

### 2.5. Mouse Hind Limb Ischemia Model and Injection of Plasmid Solutions

All animals were narcotized by intraperitoneal injection of avertin (300 µL of 2.5% solution) before surgery. Unilateral induction of hind limb ischemia was performed as previously conducted in our laboratory [8]. Briefly, skin was incised along the midline of the left hind limb hip and a. femoralis with its branches ligated distal to inguinal ligament and proximal to its popliteal bifurcation. Vessel was excised between upper and lower ligatures. Skin was closed with 5–0 silk sutures after control of hemostasis. All surgical manipulations were carried out in aseptic conditions using a binocular microscope. After completion of surgery, all animals received a 1.5-mL bolus of warm sterile saline subcutaneously to compensate blood loss and were put into a chamber until full recovery. Animals were randomized to receive experimental or control solutions. All injected plasmids were diluted in sterile saline throughout all study groups. Plasmid solution was delivered in three equal injections (50 µL each) to the anterior tibia muscle, the femoral biceps muscle and the femoral quadriceps muscle.

### 2.6. Laser Doppler Perfusion Measurement

Subcutaneous blood flow was assessed using a Laser Doppler Imaging System (Moor Instruments Ltd., Millwey, UK) as previously described [25]. Blood perfusion was measured immediately after surgery and then at days 7 and 14. Animals were narcotized by avertin intraperitoneal injection as described above. Perfusion measurements (*n* = 3–4) were made on the plantar surface of the animal’s feet, and data variability was analyzed using Moor Image Review software. Readings were taken until three subsequent runs with minimal (<10%) deviation were obtained. To account for variability among measurements, all readings were normalized against non-ischemic limbs and expressed as relative perfusion (%).

### 2.7. Muscle Harvest and Histological Analysis

At day 14 after surgery, animals were sacrificed by lethal isoflurane inhalation followed by cervical dislocation. After skin dissection, ischemic m. tibialis anterior was harvested and frozen in TissueTek medium (Sakura Finetek, Leiden, The Netherlands). Parallel frozen sections (7 µm) were prepared (using Microm HM 505E, MICROM International GmbH, Walldorf, Germany) on glass slides and stored at −70 °C until staining. For immunofluorescent analysis, m. tibialis anterior sections were fixed in ice-cold acetone for 20 min, air-dried and washed in PBS for 5 min. Slides were blocked by 10% normal donkey serum (30 min), washed and incubated overnight with primary antibodies (rat anti-mouse CD31, #553370 BD Biosciences Pharmingen, San Diego, CA, USA; rabbit anti-α-SMA antibodies, #56945 Abcam, Cambridge, MA, USA) diluted in blocking solution (1% BSA in PBS). After that, the sections were stained with Alexa Fluor^®^ 488-conjugated secondary antibody (#A21206, Thermo Scientific, Waltham, MA, USA) or with Alexa Fluor^®^ 594-conjugated secondary antibody (#A21209, Thermo Scientific, Waltham, MA, USA) (1:800) for 1 h; all slides were counterstained with DAPI (4′,6-diamidino-2-phenylindole) dye (Sigma-Aldrich, Milwaukee, WI, USA). Microphotographs of sections were taken under 200× magnification in 5 random FOVs per section using Zeiss Axio Observer A1 (Zeiss, Oberkochen, Germany). Obtained images were analyzed using ImageJ freeware (1.52q, NIH, USA). Capillary CD31+ structures and α-SMA+ blood vessels (>30 µm in diameter) were counted per FOV (ImageJ 1.52q, NIH, USA) and used to obtain mean values for sections. In total, 2–3 FOVs from 5–6 sections were analyzed for each sample to determine an average number per FOV for each animal and for the whole group.

Hematoxylin/eosin staining was used for necrotic tissue analysis. Stained muscles were photographed as described above, and necrotic/viable tissue ratio was calculated using the color threshold function in ImageJ freeware. Muscle necrosis was described as cytoplasm disruption, loss of fiber morphology, fibrosis and inflammatory cell infiltration. The obtained data were used for subsequent statistical analysis after being normalized to section area.

### 2.8. Statistical Analysis

Data were expressed as mean ± standard error of the mean (SEM) for each group. Obtained data were analyzed by Statsoft Statistica 8.0 (TIBCO Software Inc., Palo Alto, CA, USA). Statistically significant differences between 2 groups were determined by a Mann–Whitney U-test depending on the sample distribution profile. Multiple groups were compared using an ANOVA with the Bonferroni correction for level of significance where required; *p*-values < 0.05 were considered indicative of significance.

## 3. Results

### 3.1. Evaluation of Protein Expression after Delivery of Plasmid DNA Encoding HGF and VEGF165 Genes Separated by IRES

#### 3.1.1. IRES Sequences from Mammalian Genes Show Different Production of HGF and VEGF165 in HEK293T Cells

In the first round of experiments, we constructed a panel of pDNAs encoding human HGF and VEGF165 genes separated by different IRES sequences to screen for optimal gene position, choose an effective IRES and assess expressional profile of this strategy.

Translation efficacy of a protein encoded downstream to an IRES sequence is typically known to be lower than of an upstream one and the ratio of production is hardly predictable without experimental assessment. For these experiments, we used the IRES from encephalomyocarditis virus (EMCV) as a reference frequently used for mammalian expression and cloned two CMV-based plasmid constructs designated as VEGF-EMCV_IRES-HGF and HGF-EMCV_IRES-VEGF (Figure 1A). Preliminary testing in transfected HEK293T culture showed that delivery of VEGF-EMCV_IRES-HGF resulted in a very low amount of upstream VEGF165 in culture medium (1.1 ng/mL) contrasting to typically observed ≈100 ng/mL [8,26] while HGF was below the limit of detection, which basically excluded the VEGF-EMCV_IRES-HGF vector design from further application.

Using HGF-[IRES]-VEGF165 as a basis design, we cloned a panel of candidate pDNAs to screen their expression profiles. Sequences were chosen from published data on IRES efficacy in mammalian tissue [19,27]

1. EMCV of encephalomyocarditis virus (HGF-EMCV_IRES-VEGF);
2. Bip of immunoglobulin heavy chain chaperone protein gene (HGF-Bip_IRES-VEGF);
3. FGF1 of acidic fibroblast growth factor (FGF1) gene (HGF-FGF1_IRES-VEGF);
4. ElF4G of eukaryotic translation initiation factor 4G gene (HGF-ElF4G_IRES-VEGF).

Evaluation of HGF and VEGF165 expression in vitro was performed as described above. Results of the ELISA to evaluate growth factor content in medium collected after transfection are presented in Figure 1 as weight concentrations and molar ratios. Ratios of HGF/VEGF165 molar concentrations were 5.3:1 (EMCV), 10.7:1 (Bip) and 23:1 (FGF1) in samples with both proteins detected. After transfection by vector with ElF4G IRES, both proteins were close to the lower limit of detection (data not shown) so we discontinued further testing of this pDNA.

#### 3.1.2. Conditioned Medium from HGF/VEGF165-Expressing HEK293T Induces Angiogenesis in HUVEC

To evaluate angiogenic activity of HGF and VEGF165 produced by HEK293T after transfection by EMCV and Bip, we collected medium samples containing secreted proteins and transferred them to human umbilical vein endothelial cells (HUVECs) on a Matrigel-coated surface.

**Figure 2:**
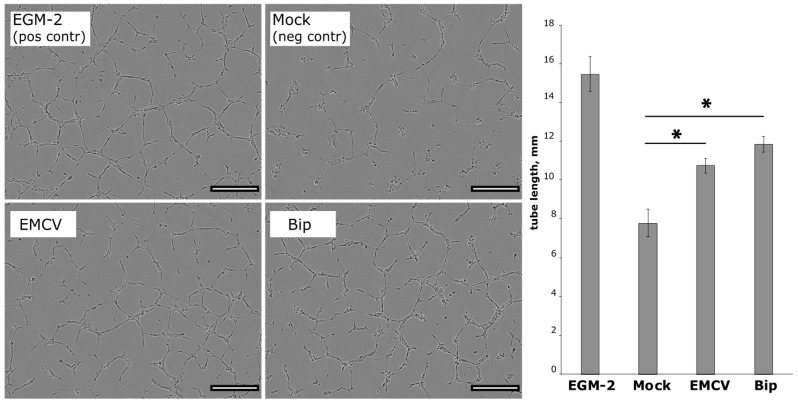
Results of tube formation assay after incubation of human umbilical vein endothelial cells (HUVECs) with culture medium from HEK293T transfected by IRES-based plasmid vectors. In Bip and encephalomyocarditis virus (EMCV), samples’ tube length was significantly higher vs. mock control (medium from mock-transfected HEK293T based on DMEM with 0.5% FBS) (* *p* = 0.0002 and *p* = 0.03, respectively); number of branching points was significantly increased in “Bip” and “EMCV” samples (* *p* = 0.0001 and *p* = 0.039 vs. mock control, respectively). Endothelial growth medium (EGM-2) was used as positive control and medium from mock-transfected HEK293T—as a negative control; Data presented as mean ± SEM. Scale bar represents 300 μm.

Tube formation after incubation of HUVECs with test samples was evaluated as mean tube length and branching point (BP) number per field of view. As expected, HUVECs showed a comparable angiogenic response after treatment with full endothelial growth medium (EGM-2) serving as a positive control (Figure 2). After HUVECs were incubated with medium from transfected HEK293T expressing HGF and VEGF165, both mean tube length and BP counts were significantly higher than in negative controls and were comparable to the positive control.

#### 3.1.3. Assessment of IRES-Based Plasmids in Skeletal Muscle Explants Demonstrates Efficient HGF but Not VEGF165 Expression

Production of human growth factors in murine skeletal muscle after pDNA injection was detected by ELISA of medium samples collected from muscle explant cultures at days 3 and 6 ex vivo. AGFs were not detected after injection of EMCV plasmid with EMCV IRES. In medium samples collected from muscle with Bip, IRES plasmid HGF was detected (718.67 ± 10.59 and 477.00 ± 8.32 pg/mL at days 3 and 6, respectively) but VEGF165 amounts were below the limits of detection. Thus, both tested IRES sequences were ineffective for simultaneous delivery of HGF and VEGF165 to mouse skeletal muscle.

### 3.2. Construction of Bicistronic Plasmids with HGF and VEGF165 Genes and Evaluation of Their Expression Activity

**Figure 3:**
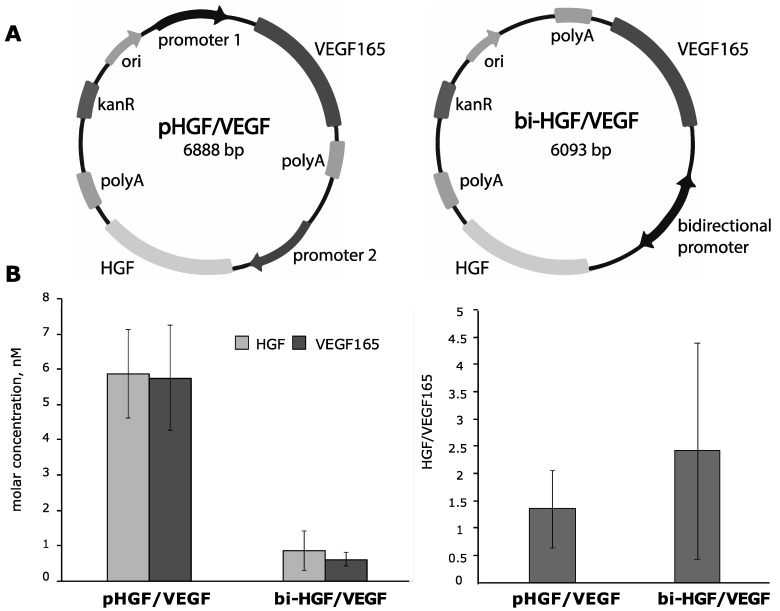
Design of bicistronic plasmids and result of expression activity analysis in HEK293T culture. (**A**) Schematic maps describing design principles of tested bicistronic vectors: pHGF/VEGF plasmid with two different promotors for HGF and VEGF165 genes and bi-HGF/VEGF plasmid with bidirectional CMV promotor. (**B**) HGF and VEGF165 concentrations and their ratio in culture medium from HEK293T cells transfected by pHGF/VEGF and bi-HGF/VEGF plasmids. Data are presented as mean ± SEM.

After failure of the IRES-based pDNAs to provide effective production of VEGF165, we switched to a strategy that exploits bicistronic vectors and constructed two new pDNAs (Figure 3A):

- pHGF/VEGF—plasmid with CMV and CAG promotors driving HGF and VEGF165 expression, respectively (Figure S2);
- bi-HGF/VEGF—plasmid with bidirectional CMV promotor for both genes.

#### 3.2.1. In Vitro Efficacy of Plasmid Vectors with Bidirectional CMV Promotor or with Independent CMV and CAG Promotors for Simultaneous Delivery of HGF or VEGF165 Genes

HEK293T cells were transfected by pHGF/VEGF or bi-HGF/VEGF and medium samples were collected 48 h after transfection. Using ELISA, we detected both VEGF165 and HGF with a molar concentrations ratio of HGF/VEGF165 of 1.38 for pHGF/VEGF and 2.47 for bi-HGF/VEGF. The highest concentration of both growth factors among all tested vectors was observed after transfection by pHGF/VEGF with independent promotors for genes of interest (6.05 ± 2.25 and 5.92 ± 2.66 nM of HGF and VEGF165, respectively). Thus, both tested bicistronic vectors showed high expression potency and ratio of HGF to VEGF165 approaching equimolar production of both AGFs.

#### 3.2.2. Conditioned Medium from HGF/VEGF165 Expressing HEK293 Renders Potent Angiogenic Effect In Vitro

HGF and VEGF165 secreted by HEK293T transfected by pHGF/VEGF and bi-HGF/VEGF were used for tube formation assay in HUVEC culture. Total tube length was significantly higher vs. the DMEM 0.5% FBS control after treatment of HUVECs by a medium from HEK293T transfected by either tested plasmid (*p* = 0.00004 and *p* = 0.006 for “pHGF/VEGF” and “bi-HGF/VEGF”, respectively). Tube formation response was also significantly higher in the “pHGF/VEGF” sample vs. the mock-transfected control (*p* = 0.019 for total tube length and *p* = 0.012 for number of master junctions).

**Figure 4:**
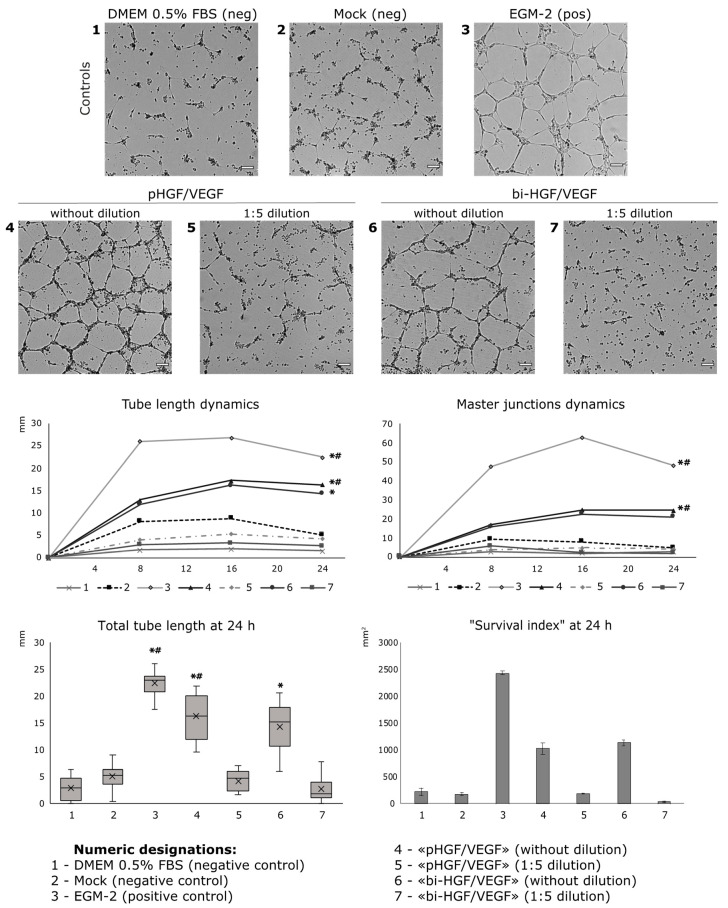
Tube formation and cell survival after incubation of HUVECs with conditioned medium from HEK293T transfected by developed bicistronic plasmid vectors and expressing HGF and VEG165. For total tubes length: * *p* < 0.0000001, * *p* = 0.00004 and * *p* = 0.006 vs. DMEM 0.5% control for EGM-2 positive control, pHGF/VEGF and bi-HGF/VEGF, respectively; *^#^ *p* = 0.0002 and *^#^ *p* = 0.019 vs. mock negative control for EGM-2 and pHGF/VEGF, respectively. For number of master junctions: * *p* < 0.0003, * *p* = 0.0035 vs. DMEM 0.5% FBS control for EGM-2 and pHGF/VEGF, respectively; *^#^ *p* = 0.00007 and *^#^ *p* = 0.012 vs. mock control for EGM-2 and pHGF/VEGF, respectively. Scale bar represents 100 μm.

At high concentrations, HGF increases the mobility and migration of endothelial cells, which may lead to the disintegration of new capillaries. This phenomenon was initially termed scattering and became the rationale for HGF being referred to as a scatter factor in early studies [28,29]. Previously, ELISA detected a relatively high amount of HGF (6.05 ± 2.25 nM for pHGF/VEGF) in culture medium from transfected HEK293T; thus, for the tube formation assay, we added two experimental groups with diluted (1:5) culture medium for both plasmids. In the latter samples (“pHGF/VEGF 1:5 dilution” and “bi-HGF/VEGF 1:5 dilution”), stable tube formation was not observed (Figure 4).

To assess survival, cells were stained with Hoechst 33342 and ethidium homodimer at 24 h of tube formation in separate wells of a culture plate. Hoechst 33342 marked the nuclei of all cells and ethidium homodimer was used to label dead cells. “Survival index” was determined after plate wells were washed to discard the dead cells using the formula ∑*_area Hoechst_* − ∑*_area ethidium homodimer_* as described in “Materials and Methods”. The “survival index” was dramatically low in the mock and DMEM 0.5% FBS negative controls and was much improved in the medium from transfected HEK293T cells, while in the EGM-2 positive control, most HUVECs were alive.

**Figure 5:**
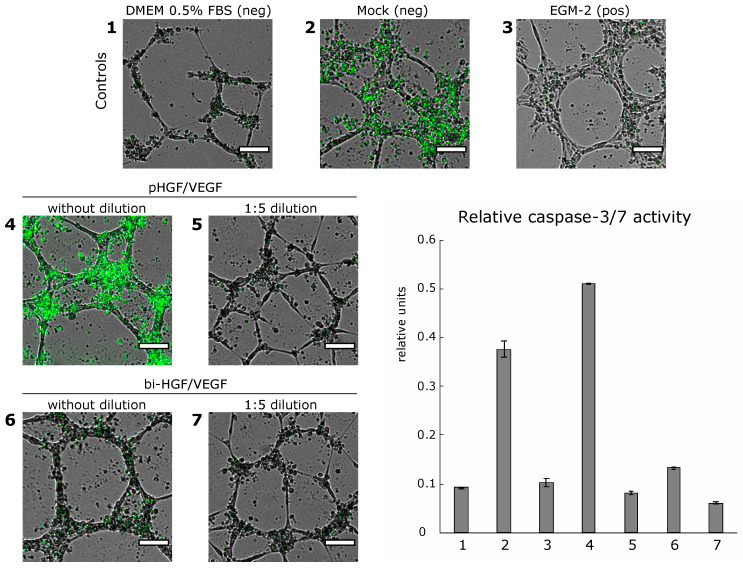
Relative caspase-3/7 activity in HUVECs visualized using a specific fluorescent substrate. Relative caspase-3/7 activity was determined by the following formula: caspase-3/7 dye sum area (green)/cells sum area. Data are presented as mean ± SEM. Scale bar represents 100 μm.

Once we encountered pro-survival effects of produced HGF and VEGF165, an evaluation of caspase-3/7 activity was addressed to study the cell death mechanism and impact of angiogenic growth factors produced after pDNA transfection. Caspase activity was assessed at the 24 h endpoint by measurement of fluorescence of a commercial caspase-3/7 substrate (Incucyte Caspase-3/7 Green Dye). The highest caspase-3/7 activity was registered in the mock control and “pHGF/VEGF” samples, while in other tested samples, it was relatively low (Figure 5). The negative DMEM 0.5% control had a very low signal of caspase-3/7, putatively due to major cessation of any cellular processes by 24 h with prominent signs of cell death and thinning of tubules due to HUVEC starvation.

#### 3.2.3. Ex Vivo Assessment of HGF and VEGF165 Production by Skeletal Muscle Explants Shows Dramatic Difference of Bicistronic Vectors

**Figure 6:**
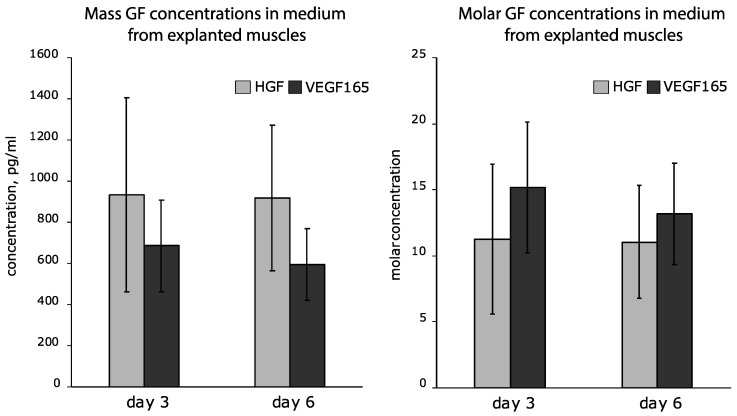
Mass and molar concentrations of human HGF and VEGF165 produced by skeletal muscle explants transfected by pHGF/VEGF plasmid. Data are presented as mean ± SEM.

Production of human AGF by murine muscle after injection of 100 µg of pDNA was detected by ELISA of medium samples collected from explant cultures at days 3 and 6 of culture. After injection of the bi-HGF/VEGF vector, we detected very high amounts of HGF, but expression of VEGF165 was below the limit of detection. Administration of pHGF/VEGF plasmid resulted in efficient transfection and production of significant amounts of both AGFs. Average concentrations of HGF and VEGF165 at days 3 and 6 after transfection by pHGF/VEGF are presented in Figure 6.

Thus, screening of seven plasmid DNA designs has yielded the optimal vector with two independent promoters (pHGF/VEGF) that provided significant amounts of both AGFs in vitro and in skeletal muscle ex vivo. The proportion of HGF/VEGF165 molar concentrations produced by muscle explants transfected by pHGF/VEGF was close to equimolar (≈1–1.2), which allowed for proceeding to animal tests in a hind limb ischemia model to assess its angiogenic efficacy.

### 3.3. Effect of Angiogenic Bicistronic Plasmid Transfer in Mouse Hind Limb Ischemia Model

To evaluate the therapeutic efficacy of pHGF/VEGF on perfusion recovery of ischemic tissue, we utilized a mouse model of hind limb ischemia. After a. femoralis ligation and excision, all animals had a comparable reduction in perfusion rate down to approx. 10% compared to the contralateral limb indicating severe ischemia. No significant deviation of baseline values on day 0 was found among study groups. After induction of unilateral ischemia, mice were distributed to experimental groups and received one of the treatments. Plasmids were delivered in three equal injections (50 μL each) to m. tibialis anterior, m. biceps femoris and m. quadriceps femoris without electroporation. After surgery, all animals received a 1.5-mL bolus of warm sterile saline subcutaneously to compensate for blood loss. The following treatments were used:

(1) “pHGF/VEGF”—single delivery of pHGF/VEGF (150 μg)*, *n* = 9;
(2) “empty vector”—negative control group with single delivery of empty pDNA backbone (commercially available pVAX2) (150 μg)*, *n* = 7;
(3) “saline”—vehicle negative control with 0.9% NaCl injection (150 μL), *n* = 8.

* All pDNAs were dissolved in 150 μL of saline.

#### 3.3.1. Single pHGF/VEGF Plasmid Injection Induces Effective Restoration of Blood Flow in Ischemic Limb

**Figure 7:**
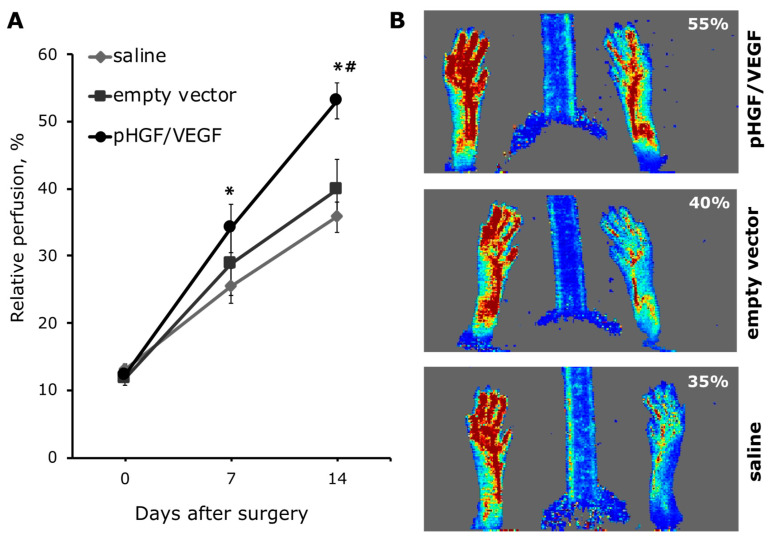
Evaluation of murine ischemic limb perfusion in study groups. (**A**) Limb perfusion graph indicating relative perfusion values; for the pHGF/VEGF group: * *p* = 0.05 and *p* = 0.006 vs. saline group at days 7 and 14, respectively; *^#^ *p* = 0.018 vs. empty vector at day 14. (**B**) Representative Laser Doppler images of subcutaneous blood flow in mice from experimental groups obtained at day 14 after ischemia induction and plasmid injection. Data are presented as mean ± SEM.

It is known that C57BL/6 mice have strong vascular repair ability, so for perfusion recovery assessment, we chose the first 14 days after surgery. Limb perfusion measurements by Laser Doppler were performed immediately after ischemia induction (day 0) and at days 7 and 14 after surgery and plasmid injection. We found that single injection of pHGF/VEGF plasmid induced significant restoration of hind limb perfusion at day 7 (34.14% ± 3.6% for pHGF/VEGF vs. 25.51% ± 2.59% in saline group; *p* = 0.04). By day 14, these differences increased even more and perfusion in the pHGF/VEGF group was 1.5-fold higher than in the saline group (53.06% ± 2.71% vs. 35.76% ± 2.20%, respectively; *p* = 0.006) and 1.33 times higher than in the empty vector (53.06% ± 2.71% vs. 39.83% ± 4.53%, respectively; *p* = 0.018). Aggregate data of perfusion dynamics and representative Doppler scans are presented in Figure 7.

#### 3.3.2. Histological Analysis of Necrosis Span in Ischemic Skeletal Muscle

Beside vivid cyanosis in the early days of the experiment and minimal trophic changes of epidermis at week 2, we observed no signs of significant necrotic changes in ischemic limbs in the experimental animals. Thus, assessment of necrosis was performed by morphometry in histological sections of m. tibialis anterior as the distal part of limb as previously described [30].

**Figure 8:**
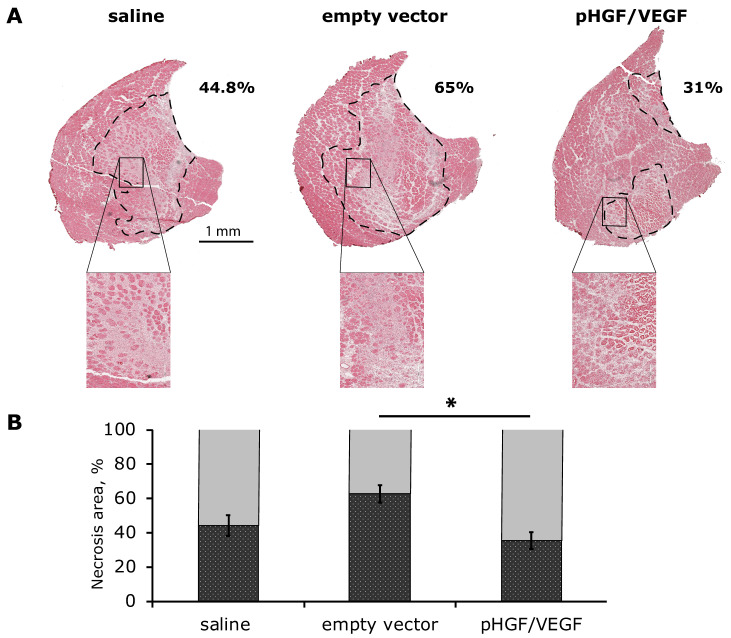
Histological analysis of necrosis in ischemic skeletal muscle at Day 14 after ischemia. (**A**) Whole-section images of hematoxylin/eosin-stained m. tibialis anterior. Necrotic tissue region of interest is marked by a dashed line; high-magnification boxes demonstrate signs of muscle necrosis including lack of nuclei, fiber loss of morphology and disruption and edema accompanied by infiltration of immune cells. (**B**) Graphical presentation of necrotic tissue span. Data are presented as mean ± SEM, * *p* < 0.05 for pHGF/VEGF vs. empty vector group.

At day 14 after plasmid administration, animals were euthanized and frozen cross-sections of m. tibialis anterior were prepared for hematoxylin and eosin staining with subsequent morphometry. In most sections, necrosis signs were prominent in the central portion of muscle where s higher degree of ischemia occurs after artery excision, indicating an adequate model of ischemia. Relative span of necrosis tissue (necrotic tissue area/total section area) was significantly higher in the empty vector group compared to pHGF/VEGF and saline negative control: 62.72 ± 5.13% vs. 35.62 ± 4.95% vs. 44.14 ± 6.00%, respectively (Figure 8).

#### 3.3.3. Increased Vascularization of Ischemic Skeletal Muscle after HGF and VEGF165 Gene Delivery

**Figure 9:**
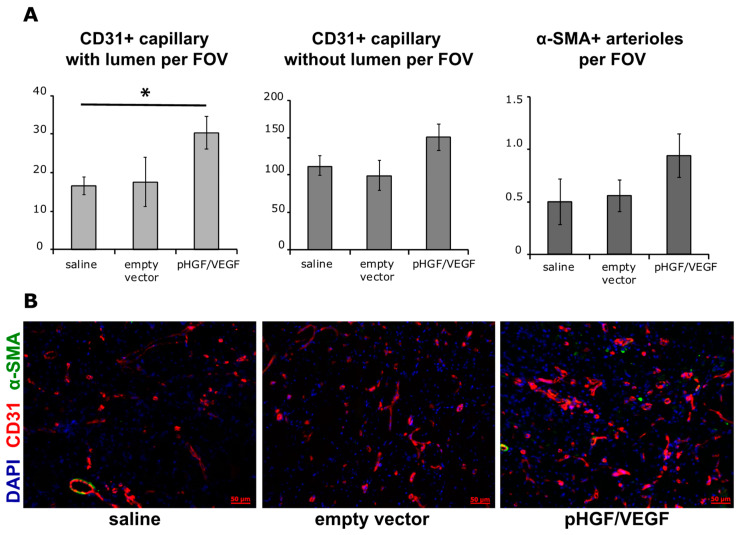
Analysis of blood vessel density in skeletal muscle sections from study group animals. (**A**) Graphical presentation of blood vessel density analysis with average group values per FOV: CD31+ capillaries with lumen, CD31+ capillaries without lumen and α-SMA+ vessels. * *p* < 0.05 for pHGF/VEGF vs. saline group. Data are presented as mean ± SEM. (**B**) Representative image of muscle section stained for CD31, α-SMA and 4′,6-diamidino-2-phenylindole (DAPI) (magnification × 200). Scale bar represents 50 μm.

Evaluation of angiogenesis was performed by counting CD31+ vessels with and without lumen (capillaries) and α-SMA+ vessels (arterioles) per FOV. A significantly higher number of CD31+ capillaries with lumen was found in pHGF/VEGF group then in saline negative control group: 30.37 ± 4.26 vs. 16.54 ± 2.30 respectively (Figure 9A). A moderate increase in CD31+ vessels without lumen was detected in pHGF/VEGF group, but this increase was not statistically significant. There were no significant differences in α-SMA+ vessels’ density in animals injected with pHGF/VEGF plasmid vs. empty vector or saline groups.

## 4. Discussion

Development of effective vectors for combined gene therapy of ischemic disease remains a challenging field, and the lack of prominent candidate drugs clearly slows progress. Our previous studies support the concept of using HGF and VEGF165, known to present a “dynamic duo”, which we characterized as prospective objects for gene delivery. We investigated their pleiotropic effects a well in relevant animal models including limb ischemia and myocardial infarction [8,9]. Both HGF and VEGF165 have already shown their efficacy, which resulted in marketing approval for gene therapy drugs based on the sole expression of these genes; in this study, we did not assess the combined approach’s superiority to each gene alone as we have already investigated this point in previous works [8,9].

The present study reports the effort to generate a single-vector non-viral drug for simultaneous delivery of VEGF165 and HGF. In our previous work, using a mixture of pDNAs bearing each factor (VEGF165 or HGF) alone, we found that equimolar production [8] was a prominent feature of this method and resulted in an impressive increase in efficacy in a limb ischemia model. Thus, a crucial criterion for the success of vector development in the present work was achieving a ratio of HGF and VEGF165 approaching 1:1.

The first part of the study clearly demonstrated a lack of expression potency in IRES-based plasmids and that, despite numerous tools that can be used for prediction of vector efficacy experimental, “trial and error” remains the only effective way to yield a candidate drug. IRESs from EMCV, Bip, FGF1 and elF4G genes were tested and demonstrated dramatic differences in protein production in eukaryotic cell cultures depending on the IRES chosen and genes positioned up- or downstream of IRES. Predicting this kind of property for a plasmid vector is marginally impossible, so we believe that our data provide a valid precaution point for developers of similar non-viral delivery systems. Analysis of published data that describe the efficacy of an IRES using reporter genes (GFP, beta-galactosidase, etc.) or non-mammalian species fails to provide sufficient cues unless you stick to the genes of interest and test the designed vector by all means possible. These results correlate with existing observations—indeed, IRES efficacy will vary depending on the position of gene around the IRES element, species, tissue and cell type and—ultimately—gene of interest [19,27].

However, a recent pilot study from a competitor group using IRES-based plasmid expressing VEGF165 and HGF reported efficacy of this candidate drug in patients (*n* = 12) with critical limb ischemia [31]. Despite impressive clinical results, little has been published on this vector expression profile and design and no data are available on the ratio of HGF and VEGF165 production after delivery to skeletal muscle.

Our study highlights the importance of experimental evaluation of vector efficacy in animal tissue as well. Only using explanted muscle culture injected with candidate plasmids, we managed to draw the final decision on discontinuing candidate vectors and switched to an independent promotor design. Interestingly, two eventual “lead” plasmids—pHGF/VEGF (driven by CMV and CAG promotors) and bi-HGF/VEGF (with both genes under a single, bidirectional modified CMV)—showed similar results in human cell culture (HEK293T) with acceptable expression of both AGFs. However, once we compared them “head-to-head” in a mouse explant model, we found that bi-HGF/VEGF completely failed to express VEGF165 yet delivered a very high amount of HGF (>6 ng/mL). Here, one may encounter a species-dependent change in the pDNA expression profile mechanism, which remains enigmatic. It is probable that a bidirectional promoter that was effective in human cells was repressed or lacked a transcription co-activator in mouse skeletal muscle, which resulted in high expression of HGF and lack of VEGF165. This piece of evidence may, on the one hand, question the relevance of mouse-to-human extrapolations, which are formally the basis of most pre-clinical studies. On the other hand, this highlights the importance of maximizing in vitro and ex vivo assessments of “lead” molecules prior to efficacy tests in animal models.

In this work, we assessed the effect of HGF and VEGF165 synthesized by HEK293T cells after transfection with experimental plasmids on the survival of HUVECs and their ability to form capillary-like structures. It is known that AGFs promote cell survival, exhibiting anti-apoptotic effects through mitogen-activated protein kinase/extracellular signal-regulated kinase (MAPK/ERK) and alpha serine/threonine-protein kinase (Akt) pathways [28]. It also was shown that HGF protects endothelial cells against hypoxic injury by inhibition of p38 MAPK and Bid/Bax and increasing expression of Bcl-2 or Bcl-xl [29]. HGF and VEGF165 promote vascular endothelial cell survival by induction of the anti-apoptotic genes Bcl-2 and A1 [30]. Activation of these signaling pathways was also shown in our previous work—treatment with VEGF165 and HGF together resulted in greater phosphorylation of ERK1/2 [8]. Our previous data also provided evidence of VEGF165 and HGF cooperatively modulating the stability of hypoxia-inducible factors (HIF-1 and -2) and regulating their target genes’ expression, many of which are related to angiogenic response in human tissues [9].

The present results show that the efficacy of tube formation in vitro depended heavily on the amount of viable cells, and AGF addition supports cell survival. To study the mechanism, we evaluated the activity of caspase-3/7. In the mock control (medium from HEK293T cells with mock transfection), cell “survival index” was low and activation of caspase-3/7 was observed, which indicates that HEK293T cells might themselves produce proapoptotic factors causing the death of HUVECs. Indeed, transfection-related stress and use of calcium for DNA delivery can induce production of apoptotic bodies and alter transfected cells’ secretome, resulting in a pro-apoptotic spectrum. In the medium collected from HEK293 transfected with the bi-HGF/VEGF plasmid, cell survival was higher than in the mock control, while the fluorescence of the cleaved caspase-3/7 substrate was insignificant, which complies with literature data showing that AGFs have anti-apoptotic properties and can suppress activation of caspase-3/7. Interestingly, in HUVECs treated with a medium from HEK293 transfected with pHGF/VEGF, significant activation of caspases was observed, exceeding that in the mock control, while cell survival was comparable to that in the “bi-HGF/VEGF” sample. It should be noted that a 3-fold higher production of both VEGF165 and HGF was observed after transfection by pHGF/VEGF compared to bi-HGF/VEGF. We can assume that at high concentrations, HGF and/or VEGF165 can stimulate the activation of caspase-3/7 and manifestation of its non-apoptotic functions, which positively affect the survival of endothelial cells and their ability to form capillary-like structures. These findings correlate with data from other authors; pro-apoptotic proteases such as caspase-3, -7, -2, -8 have also been described in non-apoptotic events, such as cell proliferation, differentiation, osteo- and myogenesis, migration, regeneration and cell signaling [32,33,34,35]. The obtained data seem to be within a novel of angiogenesis regulation and would require further investigation.

Our animal data demonstrated that pHGF/VEGF induced a rapid response to reach significant difference of perfusion vs. saline by day 7 and by day 14; this group achieved superiority over both controls (saline and empty backbone pDNA). Empty pDNA is known to induce a certain immune response, especially prominent for vectors enriched by unmethylated cytosine–guanine dinucleotide (CpG) motifs [36,37]. For angiogenesis models, this is definitely an important control insofar as DNA-driven inflammation [38] may play a role in activation of monocytes and their macrophage progenies that play a significant role in capillary sprouting [39,40]. The latter relies on macrophage ability to secrete AGFs and destabilize matrix, which facilitate endothelial cells’ migration and formation of immature vascular trees. However, the prominent superiority of pHGF/VEGF over empty DNA control provides grounds for a conclusion that it has a beneficial effect on limb perfusion besides one rendered by DNA delivery.

Surprisingly, the empty plasmid group showed an increase in ischemic muscle damage and vast necrotic tissue. We believe that this finding should be subject to further investigation as a potential clinical risk to be assessed prior to first-in-human study. We used EndoFree plasmid purification protocols, so we suggest that effects of backbone pDNA are to be evaluated. The vector used in our study was derived from pVAX, known to carry the above-mentioned CpG oligodeoxynucleotides motifs that do stimulate immune cells [37] and necrosis of tumor [41]. HGF and VEGF165 expression after injection of pHGF/VEGF clearly alleviated this negative effect due to AGFs’ antiapoptotic influence and blood perfusion increase. Another potential limitation is the relatively short (14 days) period of observation, which might be prolonged in further studies to investigate outcomes at later time-points where resolution of necrosis in empty plasmid may occur. However, the observed effects on capillary density indicate positive impact of pHGF/VEGF on angiogenic response, which typically occurs within the first 1–2 weeks after delivery. Another rationale for relatively short terms of experiment is that in the majority of plasmid, drug expression “fades” during the first week, reaching residual level by days 10–12, which we have shown previously in concordance with other studies [8,42].

Another important point for this study is that we did not apply any technique to facilitate DNA uptake in the limb ischemia model. Typically, low-voltage pulses, ultrasound or liposomes are used, but we demonstrated that pHGF/VEGF renders a significant impact on limb perfusion even after single “naked DNA” delivery by injection. We believe that in the case of limb ischemia, our approach using multiple-point injections along the limb is a more effective way to facilitate blood vessel growth and remodeling than enhancing transfection efficacy. Our colleagues have shown that multiple intramuscular injections of small amounts of a plasmid solution (muscle chipping) provide plasmid delivery to muscle fibers of equal efficacy to a per saltum injection accompanied by hydrodynamic shock [43]. Clinical protocols with non-viral gene therapy drugs for stimulation of angiogenesis generally support this concept, and due to the low immunogenicity of pDNA, one would rather perform additional sessions of drug injections rather than complicate the therapeutic system or delivery procedure.

Finally, the angiogenic response profile was notable for the significant difference in an unusual parameter, namely CD31+ blood vessels with lumen. While the other two vessel types—CD31+ capillaries without lumen and α-SMA+ arterioles—failed to reach statistical significance, this may be attributed to the experiment’s duration (14 days) and a clear trend to pHGF/VEGF superiority was obvious.

Blood vessels with a CD31+ endothelial cell lining, a lumen and not coated by mural cells (pericytes or smooth muscle cells) may contribute to improvement of skeletal muscle perfusion and their increase may result from cooperative effects of VEGF165 and HGF. This aspect deserves additional discussion and results from existing amplification of VEGF receptors (VEGFR1 and 2) and HGF receptor (mesenchymal-epithelial transition factor, c-Met) signaling cascades in endothelial cells. Once ligands bind their receptors, activation of receptor tyrosine kinases leads to downstream activation of multiple signal molecules (p38-kinase, ERK1/2, etc.) transmitting the signal down to nuclear level to increase endothelial cells’ migration and stimulate proliferation—the cornerstones of angiogenesis. Previous studies have indicated that HGF and VEGF165 may cooperatively activate the ERK1/2 pathway, resulting in increased expression of adhesion molecules (inter-cellular adhesion molecule 1 (ICAM-1), vascular cell adhesion molecule (VCAM)) and focal adhesion kinase (FAK) phosphorylation [44]. This is of particular importance insofar as HGF in combination with VEGF165 has been shown to induce assembly of tubular structures demonstrating a lumen formed by several adjoining endothelial cells. Besides prominent morphogenic potential, VEGF165 + HGF were synergistic inducers of the anti-apoptotic genes (Bcl-2 and A1) crucial for endothelial survival under severe ischemia and accompanying inflammation [30].

Overall, we can suggest that induction of blood vessel growth driven by pHGF/VEGF has a peculiar feature with formation of small blood vessels with a lumen probably contributing to observed increase of perfusion. We may also assume that by day 14 after injection of experimental pDNA, in most studied specimens, formation of a lumen preceded further taxis of pericytes or smooth muscle cells to complete blood vessel stabilization. The latter is a potent feature of HGF known to attract mural cells to capillaries formed under the influence of VEGFR activation by its ligand [45,46].

In addition to stimulation of angiogenesis, the combination of HGF and VEGF165 may have a positive pleiotropic influence of peripheral nerve function known to be impaired in limb ischemia and diabetes. Diabetic neuropathy is a painful and hardly curable lesion that emerges from disturbance of vascular and neural trophic, which eventually leads to muscle dystrophy and irreversible frailty. Due to the complementary impact of HGF and VEGF165, we may expect that this condition is a next putative indication for developed pHGF/VEGF. Our previous experience using HGF gene delivery supports its use to stimulate nerve recovery after traumatic injury [47], while transplantation of HGF-expressing cell sheets has shown the ability to restore neuronal density in ischemic muscle [25]. Thus, simultaneous delivery of these AGFs may present an effective gene therapy approach to restore both blood supply and innervation to relieve pain and reduce tissue loss.

Supplementation of HGF by VEGF165 is also a reasonable method according to recent clinical data on a candidate drug designated VM202 (also known as Engensis) being developed by Helixmith (formerly ViroMed, South Korea). VM202 is a plasmid carrying two isoforms of HGF with 728 (HGF728) and 723 (HGF723) amino acid residues intended for the treatment of cardiovascular diseases and painful diabetic neuropathy [48,49]. In a recent press-release, it has been reported to miss the primary study endpoint and perform significantly poorer than a placebo control [50]. Thus, HGF alone (even when delivered in two isoforms) may fail to reduce diabetic neuropathy, and beneficial effects of VEGF165 may provide additional therapeutic impact to overcome this hurdle.

From a clinical point of view, the importance of next-generation gene therapy drugs relying on plasmid DNA can hardly be overestimated. Application of viral vectors for non-hereditary disorders may be limited due to the risk-to-benefit ratio profile. Potential adverse effects and toxicity of adeno-, lentiviruses and adeno-associated viruses (AAV) along with their high cost of manufacture prevent their wide spread in practice [51]. Recent deaths of two infants after acute hepatotoxicity followed by sepsis in a clinical trial using AAV, which successfully passed all pre-clinical safety assessments, sparked discussion over ability to ensure viral vector safety to provide minimal risks for patients [52]. We hope that this case will not be a blow to the field that has achieved numerous impressive milestones over several decades of its growth and trial.

In contrast, plasmid DNAs have shown a favorable safety profile over time, yet efficacy of this approach requires improvement [53]. We believe that our data are important for potential development of a first-in-class combined gene therapy drug for unmet medical needs. Indeed, the first bicistronic plasmid drug for limb ischemia is yet to be marketed or to go through the clinical trials pipeline with success, so the race still has no leader.

## 5. Conclusions

The present results indicate that a single administration of pHGF/VEGF induces angiogenesis and may alleviate ischemic necrosis, yet vector effects on inflammatory response are to be investigated in further detail during a pre-clinical study of safety. We also believe that the description of the “pipeline” we used to yield a lead molecule for in vivo tests might be useful for developers of non-viral gene therapy products.

Our data indicate that the novel pHGF/VEGF vector is a highly effective and feasible plasmid for therapeutic gene delivery to skeletal muscle. The developed vector provides an attractive option for further development to address unmet medical needs—limb ischemia, peripheral neuropathy and other diseases that can be relieved by effects of HGF and VEGF165 on vascular and neuronal trophic in impaired tissues.

## Supplementary Materials

The following are available online at https://www.mdpi.com/1999-4923/12/12/1231/s1, S1: IRES sequences from EMCV, Bip, FGF1 and elF4G genes. Figure S2: pHGF/VEGF scheme.

## Abbreviations

α-SMA	α-smooth muscle actin
AAV	Adeno-associated virus
AGF	Angiogenic growth factors
Akt	Alpha serine/threonine-protein kinase
Bip	Binding immunoglobulin protein
BP	Branching point
CAG	Chicken β-actin
cDNA	Complementary DNA
c-Met	Mesenchymal-epithelial transition factor
CMV	Cytomegalovirus
DAPI	4′,6-diamidino-2-phenylindole
DMEM	Dulbecco’s Modified Eagle Medium
EEF1A1	Elongation factor 1 human alpha
elF4G	Eukaryotic translation initiation factor 4 G
EMCV	Encephalomyocarditis virus
ERK	Extracellular signal-regulated kinase
FBS	Fetal bovine serum
FGF	Fibroblast growth factor
FOV	Field of view
GFP	green fluorescent protein
HGF	Hepatocyte growth factor
HUVEC	Human umbilical vein endothelial cell
ICAM-1	Inter-cellular adhesion molecule 1
IRES	Internal ribosomal entry site
MAPK	Mitogen-activated protein kinase
pDNA	Plasmid DNA
PGK1	Phosphoglycerate kinase
RFP	Red fluorescent protein
SD	Standard deviation
SDF	Stromal-derived factor
SEM	Standard error of the mean
uPA	Urokinase plasminogen activator
VCAM	Vascular cell adhesion molecule
VEGF	Vascular endothelial growth factor
