# Supplementary material for: Therapeutic Angiogenesis by a “Dynamic Duo”: Simultaneous Expression of HGF and VEGF165 by Novel Bicistronic Plasmid Restores Blood Flow in Ischemic Skeletal Muscle
Source: Pharmaceutics. 2020 Dec 18;12(12):1231. doi: 10.3390/pharmaceutics12121231 (PMC7766676; doi:10.3390/pharmaceutics12121231)
Supplement: Supplementary file 1 [file pharmaceutics-12-01231-s001.pdf]

pharmaceutics

Supplementary Materials: Therapeutic Angiogenesis by a "Dynamic Duo": Simultaneous Expression of HGF and VEGF165 by Novel Bicistronic Plasmid Restores Blood Flow in Ischemic Skeletal Muscle

Ekaterina Slobodkina, Maria Boldyreva, Maxim Karagyaur, Roman Eremichev,

Natalia Alexandrushkina, Vadim Balabanyan, Zhanna Akopyan, Yelena Parfyonova, Vsevolod Tkachuk and Pavel Makarevich

S1. IRES sequences from EMCV, Bip, FGF1 and elF4G genes

EMCV (575 bp) from IRESite (http://iresite.org/IRESite_web.php?page=view&entry_id=140)

CCCCTCTCCCTCCCCCCCCCCTAACGTTACTGGCCGAAGCCGCTTGGAATAAGGCCGGTGTG

CGTTTGTCTATATGTTATTTTCCACCATATTGCCGTCTTTTGGCAATGTGAGGGCCCGGAAAC

CTGGCCCTGTCTTCTTGACGAGCATTCCTAGGGGTCTTTCCCCTCTCGCCAAAGGAATGCAA

GGTCTGTTGAATGTCGTGAAGGAAGCAGTTCCTCTGGAAGCTTCTTGAAGACAAACAACGT

CTGTAGCGACCCTTTGCAGGCAGCGGAACCCCCCACCTGGCGACAGGTGCCTCTGCGGCCA

AAAGCCACGTGTATAAGATACACCTGCAAAGGCGGCACAACCCCAGTGCCACGTTGTGAG

TTGGATAGTTGTGGAAAGAGTCAAATGGCTCTCCTCAAGCGTATTCAACAAGGGGCTGAAG

GATGCCCAGAAGGTACCCCATTGTATGGGATCTGATCTGGGGCCTCGGTGCACATGCTTTAC

ATGTGTTTAGTCGAGGTTAAAAAAACGTCTAGGCCCCCCGAACCACGGGGACGTGGTTTTC

CTTTGAAAAACACGATGATAAT

FGF1 IRES A (168 bp) from IRESite (http://iresite.org/IRESite_web.php?page=view&entry_id=519), EMBL (http://rfam.xfam.org/family/RF00387#tabview=tab1)

CGCTCCAGGGGAATCAGGGCATCGCCTCCTTTTCTGGGAGGACACTCCCTTCTGATGGTGA

ATGGGAACTCCCTTCCTCCTGCAGCAGCCTGCCTGCAGCTGTCCTGGTAGAACAGTGTGGA

CATTGCAGAAGCTGTCACTGCCCCAGAAAGAAAGCACCCCAGAGCC

Bip IRES (222 bp) from IRESite (http://iresite.org/IRESite_web.php?page=view&entry_id=593, http://iresite.org/IRESite_web.php?page=view&entry_id=570), EMBL (http://www.ebi.ac.uk/ena/data/view/X87949.1), Genbank (https://www.ncbi.nlm.nih.gov/nuccore/NM_005347)

AGGTCGACGCCGGCCAAGACAGCACAGACAGATTGACCTATTGGGGTGTTTCGCGAGTGTG

AGAGGGAAGCGCCGCGGCCTGTATTTCTAGACCTGCCCTTCGCCTGGTTCGTGGCGCCTTGT

GACCCCGGGCCCCTGCCGCCTGCAAGTCGGAAATTGCGCTGTGCTCCTGTGCTACGGCCTGT
GGCTGGACTGCCTGCTGCTGCCCAACTGGCTGGCAAG

<u>eIF4G IRES</u> (368 bp) from IRESite (http://iresite.org/IRESite_web.php?page=view&entry_id=548), EMBL (http://www.ebi.ac.uk/ena/data/view/D12686), Genbank (https://www.ncbi.nlm.nih.gov/nucleotide/219612)

TCTAGATGGGGGTCCTGGGCCCCAGGGTGTGCAGCCACTGACTTGGGGACTGCTGGTGGGG
TAGGGATGAGGGAGGGAGGGGCATTGTGATGTACAGGGCTGCTCTGTGAGATCAAGGGTC
TCTTAAGGGTGGGAGCTGGGGCAGGGACTACGAGAGCAGCCAGATGGGCTGAAAGTGGAA
CTCAAGGGGTTTCTGGCACCTACCTACCTGCTTCCCGCTGGGGGGTGGGGAGTTGGCCCAG
AGTCTTAAGATTGGGGCAGGGTGGAGAGGTGGGCTCTTCCTGCTTCCCACTCATCTTATAGC
TTTCTTTCCCCAGATCCGAATTCGAGATCCAAACCAAGGAGGAAAGGATATCACAGAGGA
GATC

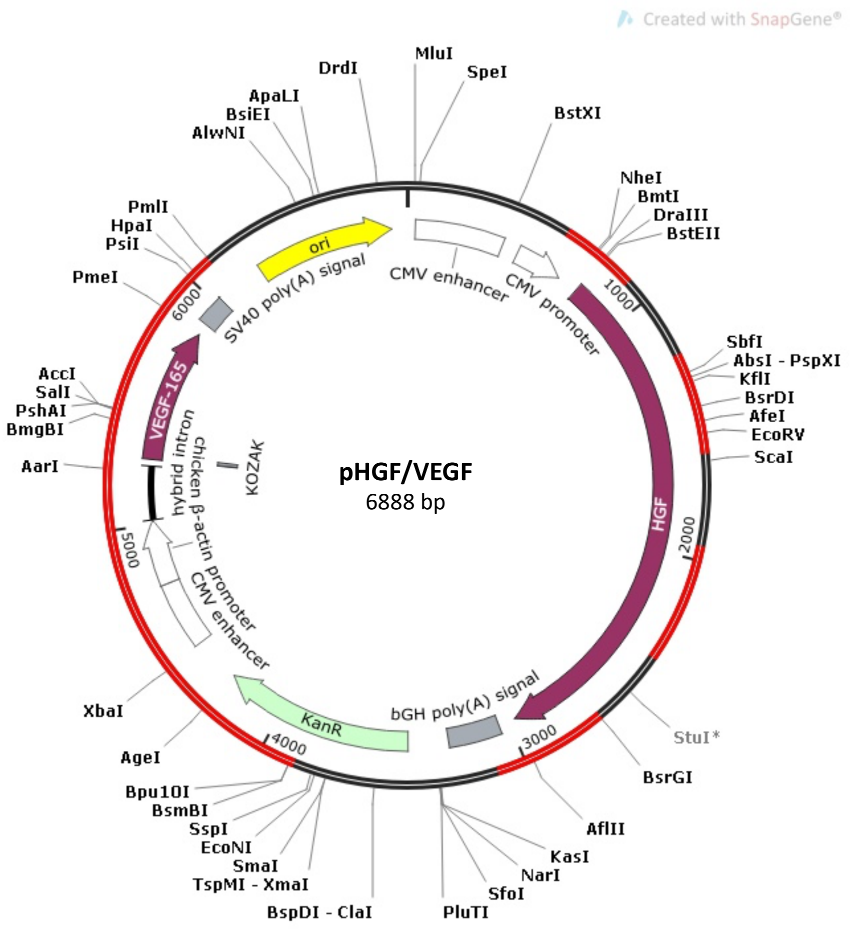

Figure S2. pHGF/VEGF scheme
